# Ramatroban as a Novel Immunotherapy for COVID-19

**DOI:** 10.37421/jmgm.2020.14.457

**Published:** 2020-07-30

**Authors:** Ajay Gupta, Kamyar Kalantar-Zadeh, Srinivasa T. Reddy

**Affiliations:** 1 Division of Nephrology, Hypertension and Kidney Transplantation and Department of Medicine, University of California Irvine (UCI) School of Medicine, United States; 2 Departments of Medicine, and Molecular and Medical Pharmacology, David Geffen School of Medicine at UCLA, Los Angeles, CA 90095, United States

**Keywords:** SARS-CoV-2 Virus, Ramatroban, COVID-19, Immune Function

## Abstract

SARS-CoV-2 virus suppresses host innate and adaptive immune responses, thereby allowing the virus to proliferate, and cause multiorgan failure, especially in the elderly. Respiratory viruses stimulate cyclooxygenase-2 (COX-2) to generate prostanoids including Prostaglandin D_2_ (PGD_2_) and thromboxane A_2_. Furthermore, PGD_2_ concentrations in the airways increase with aging. PGD_2_ action mediated via DP_2_ receptors suppresses both innate and adaptive immune responses, by inhibiting interferon-λ and stimulation of myeloid monocyte-derived suppressor cells respectively. PGD_2_ and thromboxane A_2_ actions via the TP receptors activate platelets leading to a prothrombotic state. Ramatroban, a small-molecule antagonist of DP_2_ and TP receptors, reverses viremia-associated proinflammatory, immunosuppressive5 and prothrombotic processes which are similar to those induced by SARS-Cov-2. Ramatroban, used for the treatment of allergic rhinitis in Japan for the past 20 years has an excellent safety profile. Therefore, Ramatroban merits investigation as a novel immunotherapy for the treatment of COVID-19 disease.

## Introduction

Novel coronavirus disease 2019, also known as COVID-19, is a highly infectious, rapidly spreading viral disease with very high morbidity and mortality [1]. The death rate with COVID-19 increases with advancing age, which could be due to several factors including progressive decline in immune function with aging [2]. Although SARS-CoV-2 and the 2003 SARS-CoV infections share a number of common clinical manifestations, SARS-CoV-2 virus appears to be highly efficient in person-to- person transmission and frequently cause asymptomatic infections, but the underlying mechanisms that confer the above characteristics of COVID-19 disease remain incompletely understood [3].

## Literature Review

SARS-CoV-2 and SARS-CoV are similar in cell tropism, with both targeting types I and II pneumocytes, and alveolar macrophages [3]. In mouse models of viral respiratory infections, airway epithelial cells produce prostaglandin D_2_ (PGD_2_) in an age dependent manner, with higher levels in older mice [4].There is increasing evidence that following respiratory virus infection, PGD_2_ mediates airway inflammation while suppressing the host immune response to the virus [5]. We propose the hypotheses that higher basal production of PGD_2_ in the airways of the elderly is causally linked to more severe disease with COVID-19; and that PGD_2_ suppresses the innate and adaptive immune responses to SARS-CoV-2 allowing robust unchecked viral replication and virulence. We present evidence that Ramatroban, a small molecule antagonist of PGD_2_ and thromboxane A_2_, currently approved and used in Japan for the treatment of allergic rhinitis, merits immediate investigation as a potential therapeutic agent against SARS-CoV-2.

### Respiratory viruses’ upregulate PGD_2_ production in lungs and airways, and PGD_2_ regulates host immune responses to the virus

SARS-CoV-2 was found to be capable of infecting and replicating about 3 times more robustly than SARS-CoV in ex-vivo studies of human lung tissues [3]. The first line of defense against respiratory viruses, including influenza, rhino, respiratory syncytial, SARS-CoV and SARS-CoV-2 viruses are Type III interferons, including interferon-λ (IFN-λ) [interleukin-28A/B (IL-28A/B)] [6]. In the respiratory tract, IFN-λ expression is selective to mostly respiratory epithelial cells and dendritic cells. Both in vitro and in vivo studies have demonstrated that IFN-λ is as effective as Type I interferons in anti-viral activity [7]. The role of interferons in differential replication rates has been studied. The 2003 SARS-CoV infection resulted in significant upregulation of types I (IFNβ), II (IFNγ), and III (IFNλ1, IFNλ2, and IFNλ3) IFNs in human lung tissues, but SARS-CoV-2 infection did not significantly trigger the expression of any IFN [3,8]. Recent studies have demonstrated that interferon response to viral infections is regulated by local production of prostaglandins, especially prostaglandin D_2_ [9].

Prostaglandins are known to play a key role in immune and inflammatory responses [10]. Respiratory viruses, including respiratory syncytial and SARS-CoV viruses, increase prostaglandin D_2_ (PGD_2_) in the airways in pathogen dependent manner [4,9]. Infection with respiratory syncytial virus increased PGD_2_ release by cultured human primary airway epithelial cells [9]. Intranasal administration of SARS-CoV virus to mice resulted in marked increase in PGD_2_ levels in the lungs [11]. Moreover, PGD_2_ production was elevated in nasopharyngeal samples from young infants hospitalized with RSV bronchiolitis, compared to healthy controls [9]. To the best of our knowledge PGD_2_ levels have not been examined in lung, airways or hematopoietic cells from patients with SARS-CoV or SARS-CoV-2 infections.

The increased generation of PGD_2_ in response to viral infections is primarily mediated by upregulation of COX-2, phospholipase A_2_ and PGD_2_ synthase as reviewed here. Transcriptional activation of COX-2 is mediated by multiple mechanisms. Intratracheal administration of a synthetic double stranded viral RNA (dsRNA) to mimic viral infection induced gene expression of cyclooxygenase-2 (COX-2) by direct binding to the COX-2 promoter [5]. Additionally, the nucleocapsid protein of SARS-CoV activates the expression of COX-2 by binding directly to the regulatory elements for nuclear factor-kappa B and CCAAT/enhancer binding protein [12]. HIV-2 efficiently induces COX-2 transcription in human astrocytes through regulation of NF- κB p65/relA phosphorylation and transactivation [13]. COX-2 transcription is also induced directly or indirectly by inflammatory molecules released either as a result of viral infection per se or the host response to the virus [12]. Intranasal administration of SARS-CoV virus to mice resulted in marked increase in phospholipase A_2_ expression and PGD_2_ levels in the lungs.^11^ Infection with respiratory syncytial virus up-regulated hematopoietic prostaglandin D synthase expression in cultured human primary airway epithelial cells [9]. Therefore, as reviewed here, it has been conclusively demonstrated that respiratory viruses upregulate PGD_2_ production in the airways and the lungs.

Studies over the past decade have investigated the role of PGD_2_ in regulating the innate and adaptive immune responses to the SARS-CoV viruses. PGD_2_ signals primarily through three G-protein coupled receptors, first the D-prostanoid receptor 1 (DP_1_); second, prostanoid receptor 2 (DP_2_) which was identified previously as the “chemoattractant receptor-homologous molecule expressed on Th2 cells” (CRTH2); and third, the thromboxane receptor (TP) [14,15]. PGD_2_ effects on cellular elements in the respiratory tree including pulmonary capillary endothelial cells, airway epithelial cells and cells of the innate and adaptive immune system are diverse and cell specific [4].

Werder RB, et al. have demonstrated in a neonatal mouse model of severe viral bronchiolitis that production of IFN-λ is dependent on PGD_2_/DP_2_ signaling; PGD_2_/DP_2_ antagonism decreases viral load, immunopathology, and morbidity [9]. The beneficial effects of DP_2_ blockade were associated with increased IFN-λ (IL-28A/B) expression and were lost upon IFN-λ neutralization [9]. This suggests that PGD_2_/DP_2_ antagonists may be useful antivirals for the treatment of respiratory infections including SARS-CoV-2.

**Figure 1.**
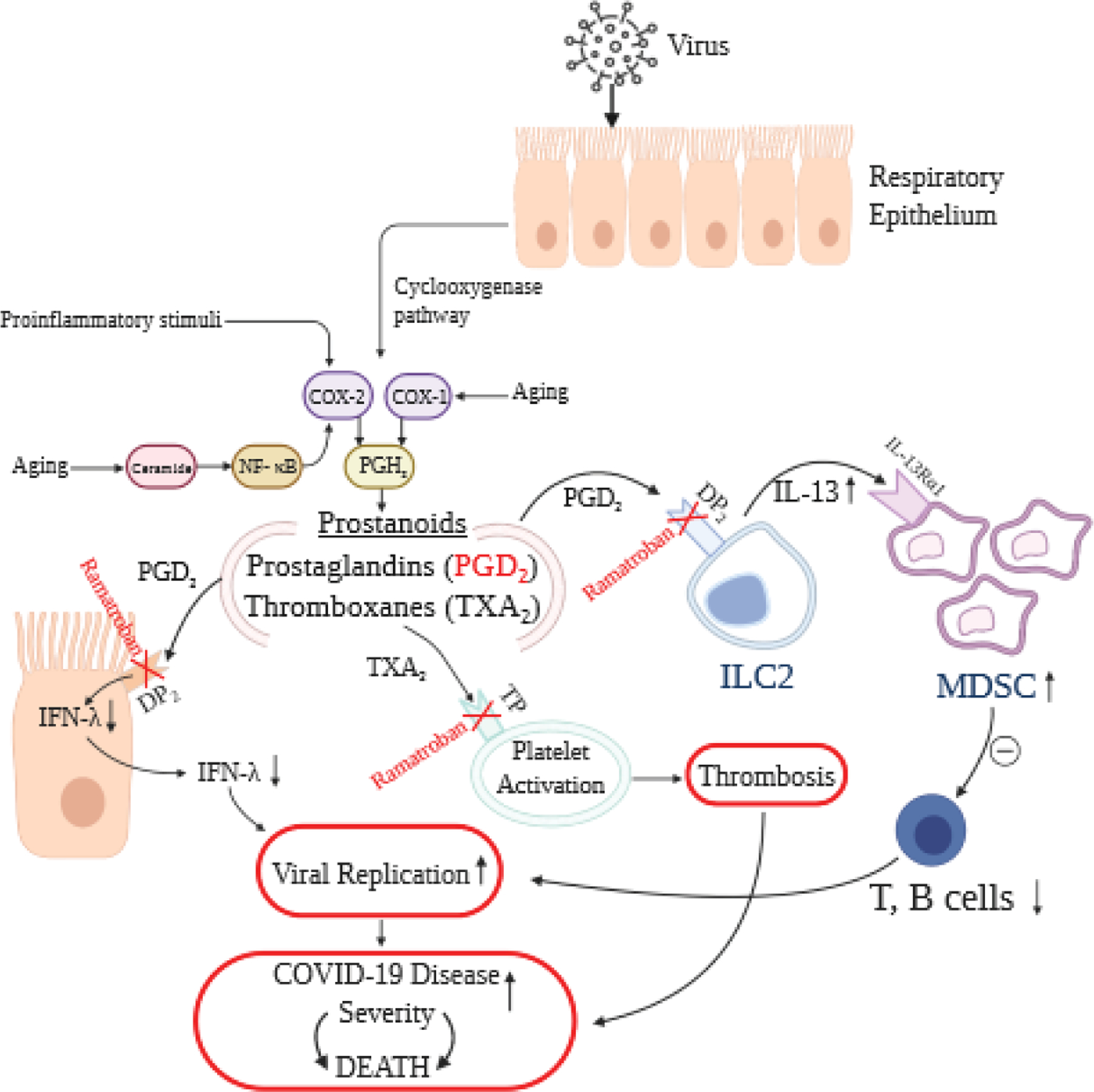
COVID-19 disease characterized by immune suppression, inflammation and a prothrombotic state.

T cells are necessary for viral clearance, and, the development of robust T cell responses in the lung requires well-functioning respiratory dendritic cells (rDC) to process and present antigens, migrate to draining lymph nodes and stimulate adaptive immunity against the virus, including cell and humoral mediated immune responses. Experimental data suggests that increase in PGD_2_ expression in mouse lungs following viral infection leads to impairment in rDC migration to mediastinal lymph nodes [4]. The production of PGD_2_ increases with aging, which results in diminished T cell responses and a more severe clinical disease in older mice infected with respiratory viruses [4]. Furthermore, PGD_2_ drives ‘group 2 innate lymphoid cells’ (ILC2) to secrete interleukin-13 (IL-13), which activates “monocytic myeloid-derived suppressor cells (M-MDSCs)” to suppress downstream immune responses [16]. Blocking the PGD_2_ pathway by a specific antagonist of the DP_2_ receptor led to a decrease in ILC2 and M-MDSC cells, demonstrating that the ILC2/M-MDSC immunosuppressive axis is partly driven by high PGD_2_ concentrations acting upon the DP_2_ receptor on ILC2 cells (Figure 1).

It is important to note that production of PGD_2_ by airway epithelial cells upon infection with respiratory viruses has salutary effects through the other PGD_2_ receptor, D-prostanoid receptor (DP_1_), in that PGD_2_/DP_1_ signaling upregulates interferon and accelerates viral clearance. There may be beneficial effects of PGD_2_/DP_1_ system in acute lung injury. PGD_2_/DP_1_ signaling tightens endothelial barrier function in lipopolysaccharide (LPS) induced acute lung injury, while DP_2_ antagonism was not harmful [17]. The biological effects of PGD_2_/DP_1_ axis in the respiratory system seem to be largely anti-inflammatory and opposed to the effects of PGD_2_/DP_2_ signaling which has deleterious effects, as stated above [9,16]. Ramatroban is a selective blocker of DP_2_ and TP receptors, and does not affect PGD_2_ signaling through DP_1_ receptor.

### PGD_2_, a potential mediator of increased morbidity and mortality in COVID-19 disease.

It is well known that during the aging process, immune functions decline, rendering the host more vulnerable to certain viruses. The morbidity and mortality from SARS-CoV-2 also increases with both aging and lymphopenia [18,19]. The mechanisms underlying this age-dependent susceptibility to viral infections are an active area of research. Stanley Perlman and colleagues have demonstrated that with aging, mice exhibit a higher basal PGD_2_ levels in the airways and lungs, with 22-months old mice exhibiting PGD_2_ levels that are 4–5 fold higher, compared to the 6-weeks old mice [4]. They further infected mice of various ages with SARS-CoV intranasally. While all 8 of the 22-month old mice died, all 14 of the 6-week old mice survived. Furthermore, increase in PGD_2_ expression in mouse lungs upon aging correlated with a progressive impairment in respiratory dendritic cell (rDC) migration to mediastinal lymph nodes resulting in diminished T cell responses and more severe clinical disease in older mice [4]. Vijay et al have subsequently demonstrated that secreted phospholipase A_2_ (PLA_2_) group IID (PLA_2_G2D) is critical in determining the impact of age on host susceptibility to SARS-CoV [11].

Furthermore, PGD_2_ via DP_2_ signaling may have a pro-inflammatory effect by increasing the production of monocyte chemoattractant protein 1 (MCP-1) and interleukin-6 (IL-6), while IL-6 may promote virus survival and/or exacerbation of clinical disease [20,21].

### Ramatroban, an antagonist of PGD_2_/DP_2_ and Thromboxane/TP axis, as a novel immunotherapeutic drug for COVID-19.

Downregulation of the innate and adaptive immune responses to respiratory viruses is mediated by PGD_2_/DP_2_ signaling, as discussed above. This suggests that a selective blockade of PGD_2_/DP_2_ signaling without blocking DP_1_, if effectively operational, can favorably accelerate viral clearance and reduce immunopathology and morbidity. Ramatroban is a potent but reversible antagonist of PGD_2_/DP_2_ receptors, while sparing the DP_1_ receptors [14,22,23]. Ramatroban is also a potent antagonist of thromboxane receptors (TP), and inhibits tumor necrosis factor and platelet activating factor induced expression and production of monocyte chemoattractant protein-1 (MCP-1), and expression of adhesion molecules in human endothelial cells, while reducing inflammation.^22^ Ramatroban enhances vascular response to acetylcholine, and has an inhibitory effect on vascular smooth muscle contraction and platelet aggregation [22]. Ramatroban reduces myocardial infarct size, and prevents neointimal formation after balloon arterial injury in hypercholesterolemic rabbits [22,24].

Ramatroban exhibits a large safety factor. The usual dose of ramatroban is 50 to 150 mg orally, twice a day. The intravenous LD_50_ values in mice and rabbits were > 600 and > 100 mg/kg respectively, while no dogs died with an intravenous dose of 250 mg/kg [22]. In the 12-months toxicity study in dogs, no toxicologically important changes were observed in any dog given up to 30 mg/kg/day of ramatroban. In this study plasma concentration of ramatroban in animals at 2-hours after oral administration of 30 mg/kg of the drug was between 11.9 to 32.7 mg/mL, while C_max_ in healthy adult male volunteers given 75 mg of ramatroban twice daily (usual clinical dose) was about 0.4 mg/mL. Accordingly, the doses tested were judged to be sufficiently high to indicate clinical safety of ramatroban in humans. As a thromboxane A_2_ antagonist, ramatroban has been used for the treatment of allergic diseases. The clinical efficacy and safety of ramatroban have been demonstrated in clinical studies as treatment of allergic rhinitis, and for the past 2 decades ramatroban (Baynas®) has been marketed in Japan for this indication. COVID-19 is characterized by a prothrombotic state including disseminated intravascular coagulopathy (DIC), thrombosis and infarctions which are associated with poor outcomes and higher mortality [25,26].The anti-platelet action of ramatroban, as a thromboxane A_2_ antagonist, could potentially reduce thrombotic events in patients with COVID-19 disease. Therefore, ramatroban by its DP_2_ antagonism can potentially help restore the IFN-λ, T and B cell responses that have been suppressed by the SARS-Co-V2 virus in COVID-19 disease, especially in the elderly; and help control inflammation by inhibiting production of IL-6. Furthermore, ramatroban, as a potent thromboxane A_2_ antagonist can potentially reduce the severity of coagulopathy, a sequela of severe COVID-19 disease.

## Discussion and Conclusion

COVID-19 disease in more severe cases is characterized by immune suppression, inflammation and a prothrombotic state (Figure 1). Prostaglandin D_2_ has been found to be a key mediator of immunosuppressive effects in animal models of SARS-CoV infection. Ramatroban selectively antagonizes the actions of thromboxane A_2_ via the TP receptors and PGD_2_ via the DP_2_ and TP receptors while sparing PGD_2_/DP_1_ signaling (Table 1). Hence, Ramatroban, holds great potential for restoring or enhancing immune function in patients with COVID-19 disease, especially in the elderly patients. Ramatroban is expected to decrease inflammation, improve endothelial function, inhibit thrombosis and improve outcomes. Ramatroban has excellent safety profile. Therefore, Ramatroban is a highly promising therapy for patients with COVID-19 infection. Urgent and fast-track clinical trials are needed to investigate the efficacy and safety of Ramatroban across different levels of severity of COVID-19 disease. This requires cooperation between scientists, industry and governmental agencies globally.

## Figures and Tables

**Table 1.** Potential effects of Ramatroban on prostanoid signaling and anticipated biological effects.

Prostanoid / Receptor [14,15]	Signal Transduction [14]	Effect of Prostanoid on Effector Cells and Cytokines [4,9,10,16]	Effect on Airway Inflammation [5,16]	Effect of Ramatroban [14,22]
PGD_2_/DP_2_	cAMP ↓ Ca ↑	IFN-λ ↓ ILC2 ↑ MDSC ↑ Dendritic cell function ↓ T & B cell function ↓ Lymphocyte count ↓	Airway inflammation to virus ↑ (chemotaxis, WBC infiltration)	Inhibits
Thromboxane A_2_/TP	IP3 ↑ Ca ↑	Activation and aggregation of platelets Arterial and venous thrombosis DIC	Airway inflammation ↑	Inhibits
PGD_2_/DP_1_	cAMP ↑	Thymic function ↑ IFN-λ↑	Airway inflammation ↓	No effect

## Abbreviations:

PGD_2_: Prostaglandin D_2_
DP_1_: D-Prostanoid Receptor 1
DP_2_: D-Prostanoid Receptor 2
TP Receptor: Thromboxane Receptor
IFN-λ: Interferon-λ [interleukin-28A/B (IL-28A/B)]
IP3: Inositol Trisphosphate ILC2, Group 2 Innate Lymphoid Cells
M-MDSC: Monocytic Myeloid-Derived Suppressor Cells
COX: cyclooxygenase
Phospholipase A_2_: (PLA_2_) Group IID (PLA_2_G2D)
rDC: Respiratory Dendritic Cell
